# A functional polymorphism in the promoter of miR-17-92 cluster is associated with decreased risk of ischemic stroke

**DOI:** 10.1186/s12920-019-0589-1

**Published:** 2019-11-08

**Authors:** Huatuo Huang, Guijiang Wei, Chunfang Wang, Yulan Lu, Chunhong Liu, Rong Wang, Xiang Shi, Jun Yang, Yesheng Wei

**Affiliations:** 1 Department of Clinical Laboratory, The Affiliated Hospital of Guilin Medical University, Guilin, 541001 Guangxi China; 2 Department of Clinical Laboratory, The Affiliated Hospital of Youjiang Medical University for Nationalities, Baise, 533000 Guangxi China; 3 Southern Medical University, Guangzhou, 510515 Guangdong China

**Keywords:** miR-17-92 cluster, Promoter, Gene, Polymorphism, Ischemic stroke

## Abstract

**Background:**

The microRNA-17-92 (miR-17-92) cluster is one of the most extensively studied miRNA clusters. Abnormal expression of the cluster has been found to play important role in different kinds of human diseases, including ischemic stroke (IS). The aim of our study was to investigate the association between three polymorphisms (rs1491034, rs9301654 and rs982873) in the promoter of the miR-17-92 cluster and risk of IS.

**Methods:**

Three hundred and ninety-eight patients with IS and 397 control subjects were included. The genotypes of the three polymorphisms were determined by Snapshot SNP genotyping assay. Relative expression of the cluster in peripheral blood mononuclear cells (PBMCs) of cases and controls were examined by quantitative real-time PCR.

**Results:**

Significant association between rs9301654 polymorphism and risk of IS were observed basing on genotype, model and allele analyses (GA vs. AA: adjusted OR = 0.63, 95% CI: 0.41~0.97, *P* = 0.037; GG vs. AA: adjusted OR = 0.23, 95% CI: 0.07~0.78, *P* = 0.018; GA + GG vs. AA: adjusted OR = 0.57, 95% CI: 0.38~0.87, *P* = 0.009; GA + AA vs. GG: adjusted OR = 0.27, 95% CI: 0.08~0.89, *P* = 0.032; G vs. A: adjusted OR = 0.58, 95% CI: 0.40~0.83). Haplotype analysis showed that TGC and TGT haplotypes were associated with decreased risk of IS (OR = 0.59, 95% CI: 0.40~0.87, *P* = 0.007 for TGC haplotype; OR = 0.21, 95% CI: 0.06~0.75, *P* = 0.009 for TGT haplotype). Importantly, we found the expression of miR-17-5p was significant higher while miR-19a-3p was significant lower in patient with IS compared with the control group (*P* < 0.01), and patients with rs9301654GG or GA genotype displayed lower level of miR-19a-3p compared with the AA genotype (*P* < 0.01).

**Conclusions:**

Our findings indicated that rs9301654 polymorphism in the promoter of miR-17-92 cluster may be associated with susceptibility of IS in the Chinese population. However, we found that rs9301654 polymorphism and its respective gene expression did not demonstrate consistent association with IS in the Chinese population. Further studies such as gene-gene interaction are warranted to reveal the role of miR-19a and its regulatory genes in the etiology of IS.

## Background

Stroke is known as a major cause of death and long-term disability worldwide, especially in less developed country [1, 2]. The incidence of stroke is still serious in China, with about 2.6 million new stroke patients and 1.6 million stroke relate death each year [3]. Ischemic stroke (IS) is the major type of stroke, which accounted for more than 80% of all stroke type. Despite the identification of risk factors such as hypertension, diabetes, age, gender and inflammatory cytokines, the etiology of the disease remains unclear. Data from family and twins researches indicated that genetic factors may also play role in the pathogenesis of IS [4–6]. In the past several years, a large numbers of IS related susceptibility genes such as growth arrest-specific transcript 5, cluster of differentiation 40, interleukin 6 and tumor necrosis factor α have been identified [7–10].

MicroRNAs (miRNAs) are a class of small, highly conserved, non-coding RNAs that play biological function by binding to the 3′ untranslated region of target mRNA. The miR-17-92 cluster, consisted by six miRNAs (miR-17, miR-18a, miR-19a, miR-19b, miR-20a and miR-92a), is among the best-studied cluster. Knock down of the cluster can cause deadly heart and lung failure and the dysmaturity of immune cells [11]. Dysregulation of the cluster was linked with different human diseases, especially cancer [12, 13].

Recently, increasing evidence have emerged to support the role of the cluster in IS pathogenesis. The level of miR-17 was found 9.9 fold higher in serum of patient with acute IS compared with healthy controls [14], and the elevated expression of miR-17 was linked to future stroke recurrence in the other study [15]. As the another important member of the miR-17-92 cluster, miR-19a, which has been previously predicted to regulate the expression of IS related genes such as tissue factor and hypoxia inducible factors, was found significant decreased in patient with IS compared with vascular risk factor controls [16]. Importantly, recent studies have reported that the cluster can promote atherosclerosis via different signal pathways [17–22]. Atherosclerosis has been widely accepted as important pathological basis of IS. These findings indicate that the miR-17-92 cluster may be potential biomarker and therapeutic target of IS.

The miR-17-92 cluster is located in an intron of non-protein-coding gene MIR17HG (miR-17-92 cluster host gene) on chromosome 13 (13q31.3) in the human genome. Previous studies have demonstrated the association between miR-17-92 promoter polymorphisms and susceptibility of several human diseases [23, 24]. However, to the best of our knowledge, no report in literature has been found on the association between promoter polymorphisms of miR-17-92 cluster and risk of IS. Hence, this study investigated whether polymorphisms in the promoter of miR-17-92 cluster are associated with IS susceptibility in a Chinese population. Moreover, the association between these polymorphisms and the expression of miR-17-92 cluster was also further analyzed.

## Methods

### Study population

The IS group included 398 patients from department of neurology of Affiliated Hospital of Youjiang Medical University for Nationalities, Baise, Guangxi, China between January 2013 and September 2016. There were 257 men and 141 women with a mean age of 62.1 ± 10.4 years in the IS group. An established diagnosis of IS was determined according to clinical manifestations and cranial magnetic resonance imaging and/or computed tomography scans. Patients with hemorrhagic, cerebrovascular malformation, cardiogenic, tumorous, hemopathy, drugs, trauma and arteritis induced stroke were excluded from the IS group. Moreover, 397 age and gender matched controls who came to the same hospital for routine medical checkup during the same period were recruited. Individuals with cerebrovascular diseases, hereditary diseases, liver diseases, kidney diseases, nervous system disease, tumors and autoimmune diseases were excluded from the control group. There were 237 men and 160 women with a mean age of 61.3 ± 9.9 years in the control group. Clinical information such as age, gender, diabetes and hypertension were obtained from medical record review of our hospital. All participants were unrelated Chinese who were consecutively selected from the same geographic region. The study protocol was approved by the Ethical Committee of Affiliated Hospital of Youjiang Medical University for Nationalities, Baise, Guangxi, China.

### DNA isolation and genotyping

Genomic DNA was extracted from eathylene diamine tetraacetic acid-anticoagulated peripheral blood by using a commercial kit (Qiangen, China). Genotyping method was described in detail previously [24, 25]. Genotyping was performed using Snapshot SNP genotyping assay. The primer sequences were as follows: for rs1491034, 5′-TGGGGTTTCTTCAGGGGATAATTG-3′ (forward) and 5′-TGTTCTGAGTGGAGGGCCCTTAT-3′ (reverse); for rs9301654 5′-TTGGGGGAGACTGGGTAAAGAT-3′ (forward) and 5′- TGTGTGGACCTCCTCCTCCT-3′ (reverse); for rs982873 5′-CCTTGTCCGGCAATCATGAAGT-3′ (forward) and 5′-CAACATTCCCAAAGTCCGTGACA-3′. The PCRs were performed in a total volume of 20 μL which contain 3.0 mmol/L Mg^2+^, 0.3 mmol/L dNTP, 1 U HotStarTaq polymerase (QIANGEN, China), 1 μL genomic DNA, 1 μL PCR primer and 1× GC-I buffer (Takara). The PCR conditions included an initial denaturation step at 94 °C for 20 s, followed by 35 cycles with 20 s of denaturation at 94 °C, 30 s of annealing at 59 °C and 1.5 min of elongation at 72 °C, followed by a final elongation step of 72 °C for 2 min. PCR products were digested with Shrimp enzyme (SAP, from Promega) and excision enzyme (EXO I, from Epicentre). An ABI PRISM 3730XL analyser (PE Applied Biosystems, Foster City, CA, USA) sequenced the PCR products. Two independent research assistants read the results with a blindness of cases and controls. In addition, approximately 10% of all samples were randomly selected to be confirmed by DNA sequencing, and the results were 100% consistent.

### Cell isolation, RNA extraction and quantitative reverse transcription PCR assay

PBMCs were isolated from peripheral blood of the IS group and the control group using a commercial kit (Lympholyte®-H Cell Separation Media, Cedarlane). Total RNA was extracted from PBMCs of the two groups using a commercial kit (Takara, Dalian, China). For examination of the miR-17-92 cluster, 3.75 μl of RNA (0.25–8 μg) was converted to cDNA using a commercial kit according to the manufacturer’s manual (Mir-X miRNA First-Strand Synthesis Kit, Takara; Cat.No.638315). Commercial primer sets of miR-17-92 cluster were obtained from Shanghai Genesky Biotechologies Inc. Quantitative reverse transcription PCR was performed using Mir-X miRNA qRT-PCR SYBR Kit (Takara, Cat. No.638313) and ABI 7500 real-time PCR machine (Applied Biosystems, CA, USA). Data were normalized using U6 snRNA as an internal control, and the relative expression of the miR-17-92 cluster was quantified using delta-delta Ct (2^-ΔΔCt^) method.

### Statistical analysis

Continuous variables (TG, LDL-C, HDL-C and TCH) were displayed as mean ± SD, and if the data is normally distributed, Student’s t-test was used; otherwise, rank-sum test was used. Categorical variables (age, gender, hypertension, diabetes) were expressed as proportions, and Chi-squared test were used to calculate these data. Hardy-Weinberg equilibrium (HWE) was calculated by chi-squared test. Logistic regression was used to evaluate the association between polymorphisms and risk of IS. Odds ratio (OR) and 95% confidence interval (CI) values were adjusted based on age, gender, hypertension, diabetes, TG, LDL-C, HDL-C and TCH. The Benjamini-Hochberg (B-H) method was applied to control false discovery rate. Haplotype and linkage disequilibrium (LD) analysis were performed by online SHEsis software [26]. Statistical power was calculated by Quanto software (Version 1.2.4). The other statistical analyses were performed using SPSS statistical software package (SPSS Inc., Chicago, IL, USA; version 17.0). A *P* value less than 0.05 was considered as statistically significant.

## Results

### Clinical characteristics of the study population

The clinical characteristics of cases and controls are shown in Table 1. There was no significant difference between the two groups based on age and gender (*P* > 0.05). The frequencies of hypertension and diabetes mellitus in the IS group were significantly higher than those in the control group (*P* < 0.001). Serum TCH, TG and LDL-C levels in the IS group were significantly higher than those of the control group (*P* < 0.001); however, the levels of serum HDL-C in the IS group were significantly lower than those of the control group (*P* < 0.001).

**Table 1:** Clinical characteristics of the ischemic stroke patients and the control group

Characteristics	Controls (*n* = 397)	Cases (*n* = 398)	*P* value
Age, (mean ± SD, years)	61.3 ± 9.9	62.1 ± 10.4	0.277
Gender,			
Male, (%)	237 (59.7)	257 (64.6)	
Female, (%)	160 (40.3)	141 (35.4)	0.156
Diabetes, (%)			
Yes, (%)	47 (11.8)	319 (80.2)	
No, (%)	350 (88.2)	79 (19.8)	< 0.001
Hypertension, (%)			
Yes, (%)	152 (38.3)	255 (64.1)	
No, (%)	245 (61.7)	143 (35.9)	< 0.001
TG, (mmol/L)	1.08 ± 0.39	1.70 ± 1.23	< 0.001
LDL-C, (mmol/L)	2.55 ± 0.47	3.03 ± 0.76	< 0.001
HDL-C, (mmol/L)	1.75 ± 0.35	1.28 ± 0.33	< 0.001
TCH, (mmol/L)	4.91 ± 0.62	5.09 ± 0.93	0.001

*SD* standard deviation, *TG* triglycerides, *LDL* low-density lipoprotein cholesterol, *HDL* high-density lipoprotein cholesterol, *TCH* total cholesterol

### Association between rs1491034, rs9301654 and rs982873 polymorphisms and risk of ischemic stroke

The genotype distribution of rs1491034, rs9301654 and rs982873 polymorphisms and risk of IS are presented in Table 2. The genotype distribution of the three polymorphisms in the IS group and the control group were in HWE, with *P* values of 0.584 and 0.283 for rs1491034, 0.163 and 0.980 for rs9301654, 0.141 and 0.737 for rs982873. Comparing with rs9301654AA genotype, the rs9301654GA and GG genotypes were associated with decreased risk of IS even after adjusted by age, gender, diabetes mellitus, hypertension, TCH, TG, HDL-C and LDL-C (GA vs. AA: adjusted OR = 0.63, 95% CI: 0.41~0.97, *P* = 0.037; GG vs. AA: adjusted OR = 0.23, 95% CI: 0.07~0.78, *P* = 0.018). Allele analysis showed that the rs9301654G allele was associated with significantly decreased risk of IS even after adjusted by age, gender, diabetes mellitus, hypertension, TCH, TG, HDL-C and LDL-C (G vs. A: adjusted OR = 0.33, 95% CI: 0.18~0.57). Moreover, we also explored the effect of the three polymorphisms on IS risk under dominant and recessive model, and we found that the rs9301654 polymorphism was associated with a significant decreased risk of IS under both dominant and recessive model even after adjusted by age, gender, diabetes mellitus, hypertension, TCH, TG, HDL-C and LDL-C (GA + GG vs. AA: adjusted OR = 0.57, 95% CI: 0.38~0.87, *P* = 0.009; GA + AA vs. GG: adjusted OR = 0.27, 95% CI: 0.08~0.89, *P* = 0.032). However, most of the associations described above lost their statistical significance after correction by multiple comparisons. Hence, further studies especially those in larger sample sizes are needed to confirm our results.

**Table 2:** Genotype distributions of the miR-17-92 promoter polymorphisms between the ischemic stroke patients and the control group

Polymorphisms	Controls *n* = 397 (%)	Cases *n* = 398 (%)	OR (95% CI)^†^	P ^†^	P_BH_
rs1491034					
TT	100 (25.2)	113 (28.4)	1.00 (Ref)		
TC	209 (52.6)	193 (48.5)	0.89 (0.56~1.41)	0.615	0.864
CC	88 (22.2)	92 (23.1)	1.16 (0.66~2.06)	0.602	0.864
TC + CC vs. TT			0.96 (0.62~1.48)	0.850	0.864
CC vs. TC + TT			1.26 (0.77~2.04)	0.356	0.864
T	409 (51.5)	419 (52.6)	1.00 (Ref)		
C	385 (48.5)	377 (47.4)	1.06 (0.80~1.40)	0.683	0.864
HWE (*P value*)	0.283	0.584			
rs9301654					
AA	235 (59.2)	280 (70.4)	1.00 (Ref)		
GA	141 (35.5)	112 (28.1)	0.63 (0.41~0.97)	0.037	0.111
GG	21 (5.3)	6 (1.5)	0.23 (0.07~0.78)	0.018	0.09
GA + GG vs. AA			0.57 (0.38~0.87)	0.009	0.068
GG vs. GA + AA			0.27 (0.08~0.89)	0.032	0.111
A	611 (77.0)	672 (84.4)	1.00 (Ref)		
G	183 (23.0)	124 (15.6)	0.58 (0.40~0.83)	0.003	0.045
HWE (*P value*)	0.980	0.163			
rs982873					
TT	149 (37.5)	167 (42.0)	1.00 (Ref)		
TC	191 (48.1)	171 (43.0)	0.91 (0.59~1.39)	0.660	0.864
CC	57 (14.3)	60 (15.0)	1.12 (0.63~2.00)	0.705	0.864
TC + CC vs. TT			0.96 (0.64~1.43)	0.838	0.864
CC vs. TC + TT			1.18 (0.69~2.01)	0.550	0.864
T	489 (61.6)	505 (63.4)	1.00 (Ref)		
C	305 (38.4)	291 (36.6)	1.03 (0.77~1.36)	0.864	0.864
HWE (*P value*)	0.737	0.141			

*OR* odds ratio, *CI* confidence interval, *HWE* Hardy-Weinberg equilibrium

^†^Adjusted by age, gender, hypertension, diabetes mellitus, TG, LDL-C, HDL-C, and TCH; P_BH_: *P* values corrected by Benjamin-Hochberg (B-H) method

### Association between rs1491034, rs9301654 and rs982873 polymorphisms and serum lipid levels in patients with ischemic stroke

We also investigated the association between rs1491034, rs9301654 and rs982873 polymorphisms and serum TCH, TG, HDL-C and LDL-C levels in the IS group, and the results are present in Table 3. However, we didn’t observe significant association between these three polymorphisms and serum TCH, TG, HDL-C, and LDL-C level in the IS group (*P* > 0.05).

**Table 3:** Association between rs1491034, rs9301654, and rs982873 genotypes and serum lipids levels in the ischemic stroke group

Polymorphisms	n	TCH (mmol/l)	TG (mmol/l)	HDL-C (mmol/l)	LDL-C (mmol/l)
rs1491034					
TT A	113	5.19 ± 0.96	1.66 ± 1.19	1.27 ± 0.29	3.13 ± 0.72
TC + CC	285	5.06 ± 0.91	1.73 ± 1.25	1.28 ± 0.34	2.99 ± 0.77
t values		1.33	−0.49	− 0.34	1.70
*P* values		0.183	0.654	0.734	0.090
rs9301654					
AA A	280	5.09 ± 0.94	1.65 ± 1.20	1.28 ± 0.33	3.04 ± 0.76
GA + GG	118	5.12 ± 0.89	1.84 ± 1.30	1.27 ± 0.32	3.02 ± 0.76
t values		−0.34	−1.35	0.30	0.28
*P* values		0.736	0.178	0.761	0.779
rs982873					
TT	167	5.15 ± 0.96	1.77 ± 1.32	1.27 ± 0.35	3.06 ± 0.79
TC + CC	231	5.06 ± 0.91	1.66 ± 1.17	1.29 ± 0.31	3.01 ± 0.73
t values		0.93	0.84	−0.54	0.27
*P* values		0.355	0.401	0.591	0.479

*TCH* total cholesterol, *TG* triglycerides, *HDL* high-density lipoprotein cholesterol, *LDL* low-density lipoprotein cholesterol

### Genotype distribution of rs9301654 polymorphism in different population

In consideration of the important of rs9301654 polymorphism in the etiology of IS, we then perform a comparison which showing genotype distribution of rs9301654 polymorphism in different populations (Table 4). The results showed that genotype distribution of rs9301654 polymorphism in our current study were significantly different from HapMap-JPT, HapMap-ASW, HapMap-GIH, HapMap-LWK, HapMap-MEX, HapMap-MKK and HapMap-TSI populations (*P* < 0.05). However, no significant different were found when comparing with HapMap-HCB, HapMap-YRI, HapMap-CHB and HapMap-CHD populations (*P* > 0.05).

**Table 4:** Genotype distribution of the rs9301654 polymorphism in different population

		Genotypes (n, %)			Alleles (n, %)		
Population	n	AA	GA	GG	A	G	Ethnic group
Our data	397	235 (59.2)	141 (35.5)	21 (5.3)	611 (77.0)	183 (23.0)	Asian
HapMap-HCB	86	56 (65.1)	24 (27.9)	6 (7)	136 (79.1)	36 (20.9)	Asian
HapMap-JPT^Δ^	172	84 (48.8)	74 (43.0)	14 (8.1)	242 (70.3)	102 (29.7)	Asian
HapMap-YRI	226	131 (58.0)	82 (36.3)	13 (5.8)	344 (76.1)	108 (23.9)	African
HapMap-ASW^Δ^	98	43 (43.9)	44 (44.9)	11 (11.2)	130 (66.3)	66 (33.7)	African
HapMap-CHB	82	50 (61.0)	30 (36.6)	2 (2.4)	130 (79.3)	34 (20.7)	Asian
HapMap-CHD	170	112 (65.9)	50 (29.4)	8 (4.7)	274 (80.6)	66 (19.4)	Asian
HapMap-GIH^Δ^	176	134 (76.1)	42 (23.9)	–	310 (88.1)	42 (11.9)	Asian
HapMap-LWK^Δ^	180	130 (72.2)	42 (23.3)	8 (4.4)	302 (83.9)	58 (16.1)	Asian
HapMap-MEX^Δ^	100	88 (88.0)	11 (11.0)	1 (1.0)	187 (93.5)	13 (6.5)	North America
HapMap-MKK^Δ^	286	132 (46.2)	126 (44.1)	28 (9.8)	390 (68.2)	182 (31.8)	African
HapMap-TSI^Δ^	176	134 (76.1)	42 (23.9)	–	310 (88.1)	42 (11.9)	European

*HCB* Han Chinese in Beijing, China, *JPT* Japanese in Tokyo, Japan *YRI* Yoruba in Ibadan, Nigeria, *ASW* African ancestry in Southwest USA, *CHB* Han Chinese in Beijing, China, *CHD* Chinese in Metropolitan Denver, Colorado, *GIH* Gujarati Indians in Houston, Texas, *LWK* Luhya in Webuye, Kenya, *MEX* Mexican ancestry in Los Angeles, California, *MKK* Maasai in Kinyawa, Kenya, *TSI* Toscans in Italy

^Δ^: Comparing with our present data, *P* < 0.05

### Haplotype analysis of rs1491034, rs9301654 and rs982873 polymorphisms and risk of ischemic stroke

**Fig. 1:**
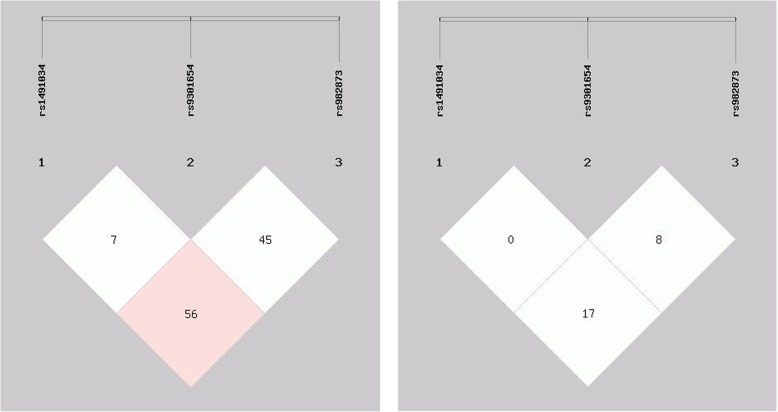
Linkage disequilibrium test of the three polymorphisms. The rs1491034 polymorphism was in linkage disequilibrium (LD) with rs982873 (D’ = 0.56, r2 = 0.17), and the rs9301654 polymorphism was in low LD with the rs982873 (D’ = 0.45, r2 = 0.08) polymorphism

Haplotype analysis was performed, and the possible eight haplotypes are listed in Table 5, and the linkage disequilibrium result are showed in Fig. 1. We found that the rs1491034 polymorphism was in low linkage disequilibrium (LD) with rs982873 (D’ = 0.56, *r*^2^ = 0.17), and the rs9301654 polymorphism was in low LD with the rs982873 (D’ = 0.45, *r*^2^ = 0.08) polymorphism. Furthermore, we found that TGC and TGT haplotypes were associated with decreased risk of IS (OR = 0.59, 95% CI: 0.40~0.87, *P* = 0.007 for TGC haplotype; OR = 0.21, 95% CI: 0.06~0.75, *P* = 0.009 for TGT haplotype).

**Table 5:** Haplotype analysis of the three polymorphisms with risk of ischemic stroke

Haplotypes	Controls 2n = 794 (%)	Cases 2n = 796 (%)	OR (95% CI)	*P* value
C A T	277 (34.9)	277 (34.8)	1.00 (Ref)	
C A C	25 (3.2)	30 (3.7)	1.20 (0.69~2.09)	0.520
C G C	39 (4.9)	30 (3.7)	0.77 (0.47~1.27)	0.307
C G T	43 (5.4)	40 (5.0)	0.93 (0.59~1.48)	0.759
T A C	154 (19.4)	180 (22.7)	1.17 (0.89~1.53)	0.261
T A T	156 (19.6)	185 (23.2)	1.19 (0.91~1.55)	0.216
T G C	86 (10.8)	51 (6.4)	0.59 (0.40~0.87)	0.007
T G T	14 (1.8)	3 (0.4)	0.21 (0.06~0.75)	0.009

*OR* odds ratio, *CI* confidence interval

### Rare genotypes of the rs9301654 polymorphism were associated with the expression of the miR-17-92 cluster

**Fig. 2:**
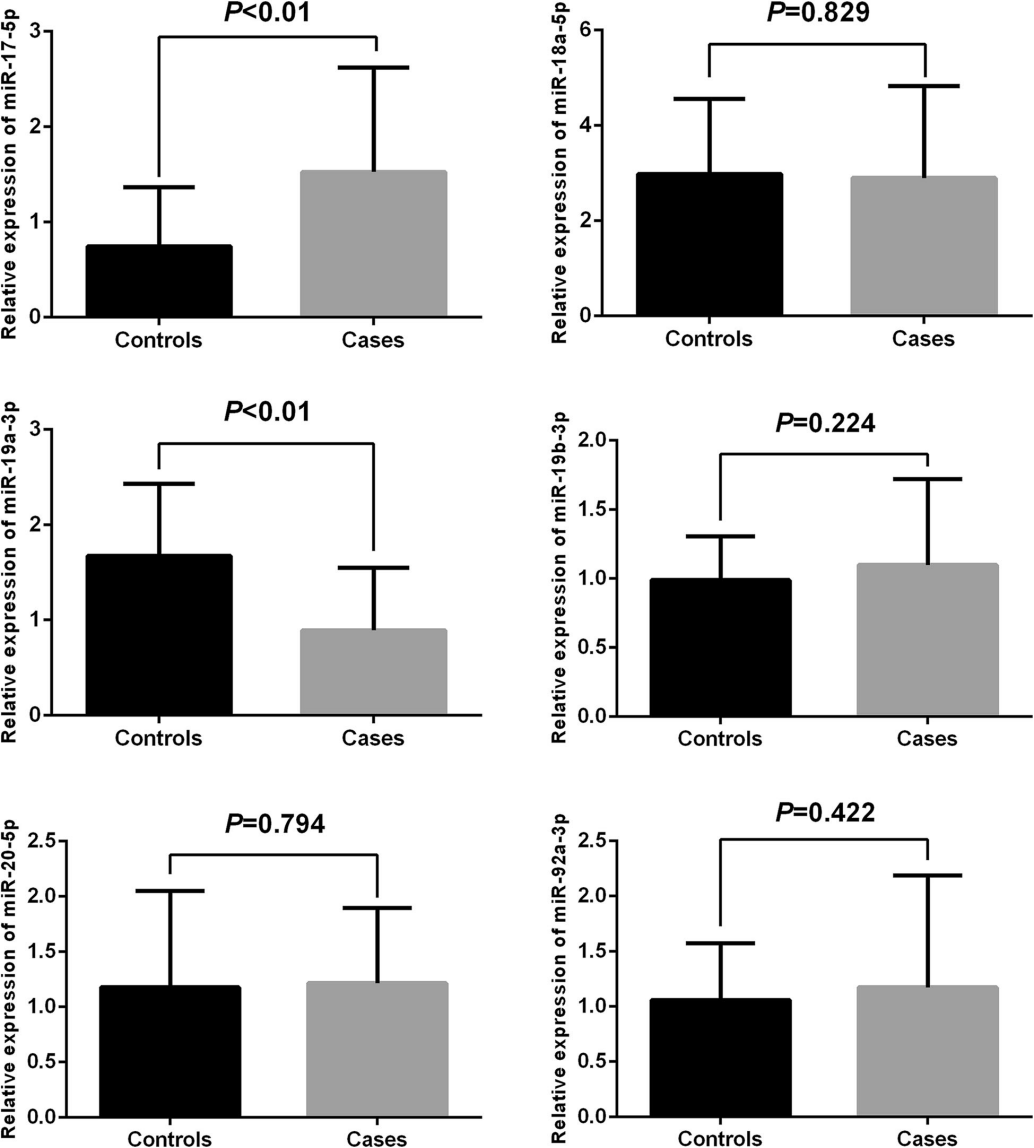
Relative expression of the miR-17-92 cluster among ischemic stroke patients (*n* = 60) and the control group (*n* = 60) in peripheral blood mononuclear cells. The data of relative expression of the miR-17-92 cluster are showed as mean ± standard deviation

**Fig. 3:**
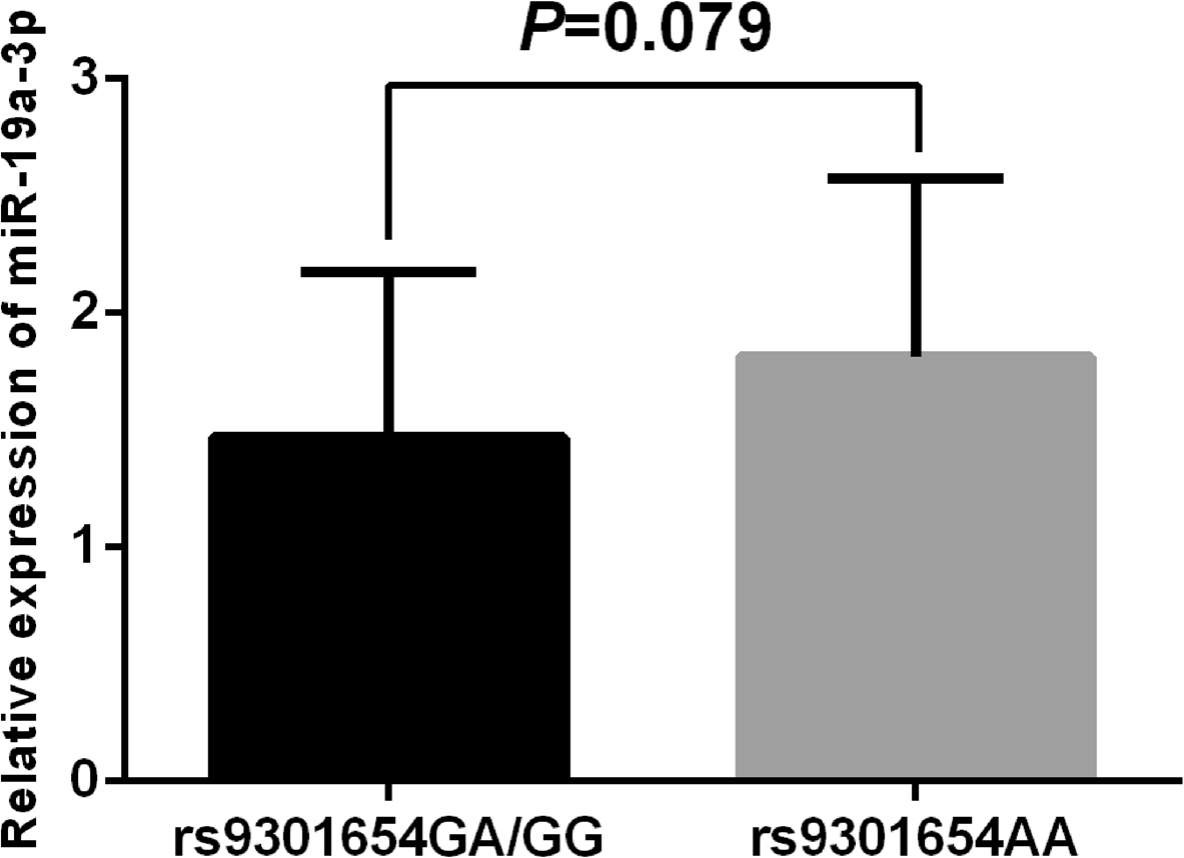
**a** The association between rs9301654 polymorphism and the expression of miR-19a in ischemic stroke patients. Patients carrying rs9301654 GA or GG genotype (*n* = 18) displayed a significant lower level of miR-19a as compared with those carrying rs9301654AA genotype (*n* = 42); **b** The association between rs9301654 polymorphism and the expression of miR-19a in the control group (*n* = 24 for GA/GG; *n* = 36 for AA). The level of miR-19a showed no different in the control group between genotypes of the rs9301654 polymorphism

In view of the positive data we obtained above, we further accessed whether the rs9301654 polymorphism will influence the expression of miR-17-92 cluster. Relative expression of the miR-17-92 cluster in the control group and the IS group were examined and we found that the relative expression of miR-17-5p was significant higher while the miR-19a-3p was significant lower in patient with IS compared with the control group (Fig. 2; both *P* < 0.01). In particular, after a systematic comparison between rs9301654 genotypes and the expression of the miR-17-92 cluster, we found that patients carrying rs9301654 GA or GG genotype displayed a significant lower level of miR-19a-3p as compared with those carrying rs9301654AA genotype (Fig. 3; *P* < 0.01). Moreover, the association between rs9301654 polymorphism and the expression of miR-19a-3p in the control group was also further analyzed. We found that controls with rs9301654GA or GG genotype also displayed lower level of miR-19a-3p than those with AA genotype, however, the difference was not significant (*P* = 0.079).

## Discussion

To our knowledge, this is the first research to explore the association between rs1491034, rs9301654 and rs982873 polymorphisms and risk of IS. The results of our study suggested that GA and GG genotypes of rs9301654 polymorphism were associated with significant decreased risk of IS. Additionally, we found that the GA and GG genotypes of rs9301654 polymorphism were associated with lower levels of miR-19a-3p compared with the AA genotype in the IS group. This study has more than 80% power to access the association between rs1491034, rs9301654 and rs982873 polymorphisms and risk of IS assuming OR of 1.6 and α of 0.05 under dominant, recessive and log-additive model (77.63, 84.46 and 99.58% for rs1491034 respectively; 90.66, 37.47 and 98.50% for rs9301654 respectively; 87.21, 70.89 and 99.56% for rs982873 respectively). Our results indicate that rs9301654 polymorphism in the promoter of miR-17-92 cluster may be associated with IS in the Chinese population.

As to the association between genetic polymorphisms and the susceptibility of IS, most of previous studies have focus on protein encoding genes. In the past few years, the association between miRNA gene related polymorphisms and risk of IS became hotpot of research [25, 27–29]. In our recent study, we found the rs4705342 polymorphism in the promoter region of miR-143/145 gene was associated with decreased risk of IS, with rs4705342CC and TC genotypes associated with decreased risk of IS compared with rs4705342TT genotype (CC VS. TT: adjusted OR = 0.53, 95% CI, 0.34–0.83, *P* = 0.006; TC VS. TT: adjusted OR = 0.74, 95% CI, 0.57–0.97, *P* = 0.03) [30]. Two studies have invested the association between miR-17-92 promoter polymorphisms and the susceptibility of human diseases [23, 24]. In one of the study, Sun et al. [23] found that rs9588884 and rs982873 polymorphisms in the promoter of miR-17-92 cluster were associated with significant decreased risk of colorectal cancer (For rs9588884: GG vs. CC: adjusted OR = 0.46, 95% CI, 0.35–0.62; dominant model: adjusted OR = 0.72, 95% CI, 0.59–0.86; recessive model: adjusted OR = 0.53, 95% CI, 0.40–0.69; For rs982873: CC vs. TT: adjusted OR = 0.60, 95%CI, 0.46–0.80; recessive model: adjusted OR = 0.62, 95% CI, 0.49–0.80). In the other study, Wang et al. [24] reported that rs9515692 polymorphism in the promoter of miR-17-92 cluster was associated with decreased risk of systemic lupus erythematosus (CT vs CC: OR = 0.65, 95%CI, 0.46–0.92, *P* = 0.014; CT + TT vs CC: OR = 0.64, 95%CI, 0.46–0.90, *P* = 0.009; T vs C: OR = 0.69, 95%CI, 0.52–0.92, *P* = 0.010). However, to our knowledge, no study has been conducted to invest the association between rs1491034, rs9301654 and rs982873 polymorphisms in the promoter miR-17-92 cluster and risk of IS. In our present study, we found that the GA and GG genotypes of rs9301654 polymorphism, and the TGC and TGT haplotypes were associated with significant decreased risk of IS. Our findings suggested that rs9301654 polymorphism in the promoter of miR-17-92 cluster may be associated with the susceptibility of IS, although validation studies need to be carried out especially in different ethnic group in the near future.

The miR-17-92 cluster is a multifunction miRNA cluster which plays crucial role in various biological function, such as normal development of heart and lung [11], and human diseases such as cancer [31] and systemic lupus erythematosus [24]. As it is known to all that atherosclerosis is important pathological basis for atherosclerosis related diseases, such as coronary artery disease and IS. Recently, increasing evidence came to support the role of miR-17-92 cluster in pathogenesis of atherosclerosis. As a key regulator for adaptation to hypoxia in cells, hypoxia inducible factors (Hif) is an important regulator of monocyte to macrophage differentiation [22]. The differentiation is crucial in physiological, and also in the pathological progress of atherosclerosis. Interestingly, upregulation of miR-17 and miR-20a were found to associated with inhibit Hif mRNA and protein expression, this finding indicated that these two miRNAs may play role in atherosclerosis progression [22]. Moreover, increased expression of miR-19a can induce the expression of chemokine C-X-C motif ligand 1 (CXCL1) in mildly oxidized low-density lipoprotein–stimulated endothelial cells [18], and CXCL1 is important in the recruitment of atherogenic monocyte in the initiation of atherosclerosis [18]. It is accepted that macrophage cholesterol accumulation and foam cells formation are two pivotal pathological process of atherosclerosis. Lv et al. reported that miR-19b can promote macrophage cholesterol accumulation and foam cells formation by specially targeting at the 3′ untranslated region of ATP-binding cassette transporter A1 [19]. In the other study, miR-19b was correlated with atherosclerosis by inducing endothelial cell dysfunction via suppression of peroxisome proliferator activated receptor γ coactivator 1α [20]. Furthermore, miR-92a can negatively regulate the expression of atherosclerosis protection factors krüppel-like factor 4 (KLF4) in arterial endothelium, and knock-down of miR-92a can partial reduce expression of proinflammatory markers such as monocyte chemotactic protein 1, vascular cell adhesion molecule-1, E-Selectin and endothelial nitric oxide synthase in vitro, which was attributable to increased KLF4 expression [32]. Taken together, these evident demonstrate that the miR-17-92 cluster may play crucial role in IS and may be a therapy target for the disease.

In this study, relative expression of the miR-17-92 cluster in PBMCs of IS patients and the control group were examined, and we found the relative expression of miR-17-5p was significant increased while miR-19a-3p was significant decreased in patients with IS compared with the control group. Several studies have invested the association between these two miRNAs and IS. In one of the study, Wu et al. found that serum miR-17-5p levels were 9.9-fold higher in patients with acute IS compared with healthy controls, and serum miR-17-5p was suggested as a promising biomarker for diagnosis of acute IS [14]. In the other study, significant increased expression of miR-17 was even linked to future stroke recurrence [15]. As to miR-19a, Jickling et al. [16] reported that the expression of miR-19a was significant decreased in peripheral blood cells of acute IS patients when comparing with vascular risk factor controls. Our findings were consistent with these previous reports. These finding suggest that miR-17 and miR-19a may be potential biomarkers and therapy targets of IS. It has been reported that polymorphisms in the promoter of a certain gene sometimes may be associated with variations in the level of transcription and expression of the mutation gene. In this study, we found rs9301654 polymorphism in the promoter of the miR-17-92 cluster was associated with decreased risk of IS. We speculated that the rs9301654 polymorphism may be associated with the expression of the miR-17-92 cluster and thus participating in IS progression. Therefore, we further investigated the association between different genotype of rs9301654 and the expression of the miR-17-92 cluster, and we found that patients carrying rs9301654 GA or GG genotype displayed significant lower level of miR-19a-3p as compared with those of rs9301654AA genotype. This result supported the assumption that rs9301654 polymorphism in the promoter of the miR-17-92 cluster is associated with gene expression. However, we note that rs9301654 polymorphism in the promoter of miR-17-92 cluster and its respective gene expression did not demonstrate consistent association with IS in the Chinese population. Further studies such as gene-gene interaction are warrant to reveal the role of miR-19a and its regulatory genes in the etiology of IS.

In the present study, control subjects with rs9301654GA or GG genotype also have lower level of miR-19a-3p than those of AA genotype, however, the different was not significant. There are several explanations for this phenomenon. As it is known to all, the expression of a certain gene can be regulated by polymorphism in promoter of the gene. At the same time, polymorphism in the promoter of a gene is not the only factor that could affect gene expression. A large number of previous studies have provided us evidences. Protein encoding gene such as placental growth factor, transcription factor E2F1 and endostatin [33–35], and long noncoding RNA (lncRNA) such as lncRNA MEG3 and LncRNA H19 [36, 37], and short hairpin RNA (shRNA) such as adenine nucleotide translocase 2 shRNA [38], have been reported to associated with abnormal expression of miR-19a in disease progression. Under normal circumstances, the normal expression of these genes does not affect the expression of miR-19a. However, during some pathologic process, abnormal expression of these genes can further down-regulate the expression of miR-19a based on the down regulatory effect of the rs9301654 polymorphism. Hence, a gene-gene interaction analysis will better reveal the roles of miR-19a and its regulatory genes in the etiology of IS in the near future.

Although the results we got were promising, several limitations should also be consideration. On the one hand, as our study was designed as a hospital-based study, thus, the possibility of selection bias cannot be eliminated, and the results we got may not be a good representative of general public. On the other hand, as it is widely accepted that genetic polymorphisms may vary in different cohorts of population, and the study population we choose were all Chinese, thus, the results we got may not be able to directly expand to other ethnic group. As is shown in the Table 4, we found that the genotype distribution of rs9301654 was significant different from HapMap-JPT, HapMap-ASW, HapMap-GIH, HapMap-LWK, HapMap-MEX, HapMap-MKK, HapMap-TSI population, however, were no significant different from HapMap-HCB, HapMap-YRI, HapMap-CHB and HapMap-CHD population. Moreover, the relatively small sample size of our present study may also a limitation, which may result in insufficient statistical power to access the relationship between polymorphisms in miR-17-92 cluster and the risk of IS.

## Conclusion

In this study, we report for the first time that rs9301654 polymorphism in the promoter of miR-17-92 cluster may be associated with the susceptibility of IS, which may provide some evidence for the etiology of IS and may be a potential biomarker and therapeutic target for the disease. However, we found that rs9301654 polymorphism and its respective gene expression did not demonstrate consistent association with IS in the Chinese population. Thus, further studies such as gene-gene interaction are warranted to reveal the role of miR-19a and its regulatory genes in the etiology of IS.

## Data Availability

The datasets used and analyzed in our current study are available from corresponding author for all reasonable request.

## Abbreviations

CI: Confidence interval
CXCL1: Chemokine C-X-C motif ligand 1
HDL: High-density lipoprotein cholesterol
Hif: Hypoxia inducible factors
HWE: Hardy-Weinberg equilibrium
IS: Ischemic stroke
KLF4: Krüppel-like factor 4
LD: Linkage disequilibrium
LDL: Low-density lipoprotein cholesterol
miR-17-92: microRNA-17-92
miRNAs: MicroRNAs
OR: Odds ratio
PBMCs: Peripheral blood mononuclear cells
SD: Standard deviation
TCH: Total cholesterol
TG: Triglycerides
