# Correction to: A functional polymorphism in the promoter of miR-17-92 cluster is associated with decreased risk of ischemic stroke

**DOI:** 10.1186/s12920-019-0631-3

**Published:** 2019-12-02

**Authors:** Huatuo Huang, Guijiang Wei, Chunfang Wang, Yulan Lu, Chunhong Liu, Rong Wang, Xiang Shi, Jun Yang, Yesheng Wei

**Affiliations:** 1 Department of Clinical Laboratory, The Affiliated Hospital of Guilin Medical University, Guilin, 541001 Guangxi China; 2 Department of Clinical Laboratory, The Affiliated Hospital of Youjiang Medical University for Nationalities, Baise, 533000 Guangxi China; 3 Southern Medical University, Guangzhou, 510515 Guangdong China

**Correction to: BMC Med Genomics (2019) 12:159**

**https://doi.org/10.1186/s12920-019-0589-1**

Following publication of the original article [1], it was reported that during the production process, Fig. 3b was omitted from the final article. The complete Fig. 3 is supplied in this correction. The original article [1] has been corrected.

**Fig. 3:**
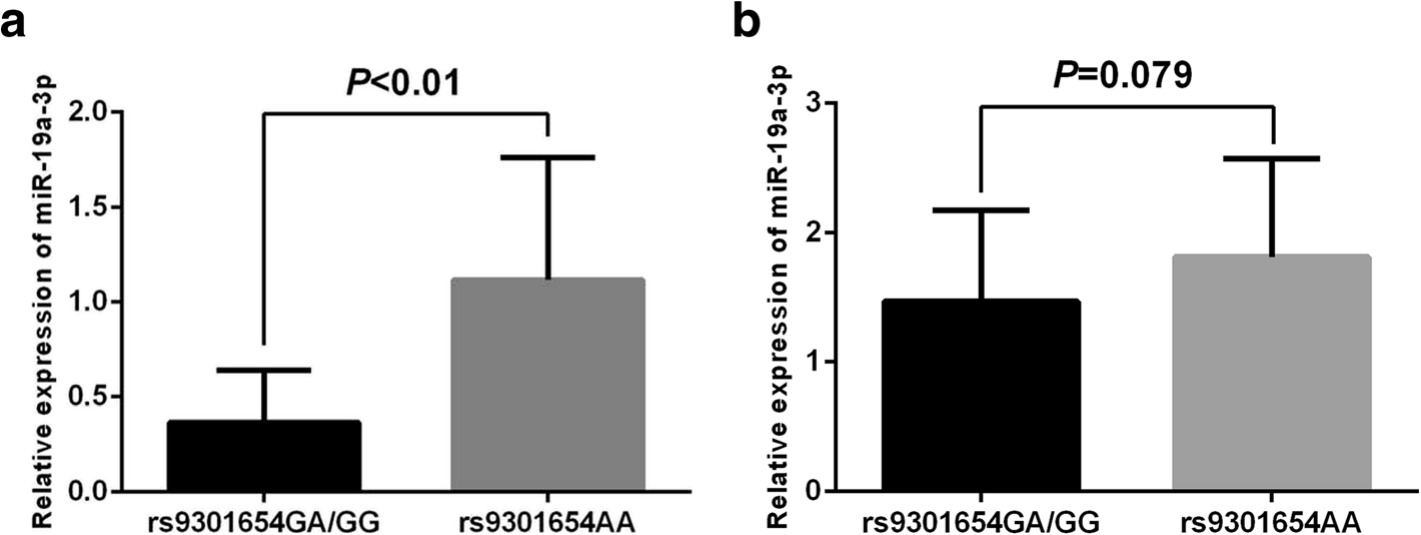
(**a**) The association between rs9301654 polymorphism and the expression of miR-19a in ischemic stroke patients. Patients carrying rs9301654 GA or GG genotype (n=18) displayed a significant lower level of miR-19a as compared with those carrying rs9301654AA genotype (n=42); (**b**) The association between rs9301654 polymorphism and the expression of miR-19a in the control group (n=24 for GA/GG; n=36 for AA). The level of miR-19a showed no different in the control group between genotypes of the rs9301654 polymorphism.
